# Irreversible electroporation ablation with bipolar electrodes: ultrasound findings of ablation zones

**DOI:** 10.2478/raon-2026-0022

**Published:** 2026-04-16

**Authors:** Linyu Zhou, Qiang Chen, Ju Li, Chengyue Zhang, Shengyong Yin, Min Xu, Tian’an Jiang

**Affiliations:** 1 Department of Ultrasound, The First Affiliated Hospital, Zhejiang University School of Medicine, Hangzhou, China; 2 Zhejiang Curaway Medical Technology Co., Ltd, Hangzhou, China; 3 The First Affiliated Hospital, Key Laboratory of Combined Multi-Organ Transplantation, Ministry of Public Health, Zhejiang University School of Medicine, Hangzhou, China; 4 Key Laboratory of Pulsed Power Translational Medicine of Zhejiang Province, Hangzhou, Zhejiang Province, China

**Keywords:** irreversible electroporation, ultrasound, contrast-enhanced ultrasound, liver, animal experiments

## Abstract

**Background:**

This study aimed to explore the ultrasound and contrast-enhanced ultrasound (CEUS) imaging characteristics and dimensions of ablation lesions after irreversible electroporation of the swine liver and to determine which imaging modality is more suitable for post-procedural follow-up by correlating imaging findings with histopathology.

**Materials and methods:**

Irreversible electroporation procedures were conducted on three swine with single bipolar electrodes. All procedures were carried out after laparotomy. Twenty-four ablation zones were created under ultrasound guidance. Ultrasound and CEUS evaluations were performed immediately and 48 h after irreversible electroporation. Liver specimens were harvested 48 h after irreversible electroporation for histopathological analysis.

**Results:**

The ablation area appeared as a hypoechoic, well-defined lesion on ultrasound and showed no enhancement on CEUS immediately after irreversible electroporation. At 48 h, the ablation zone appeared as an inhomogeneous hyperechoic area with a hyperechoic margin and blurred boundaries on ultrasound. CEUS clearly delineated the boundary of the ablation zone and demonstrated centripetal enhancement. The dimensions of the ablation area measured on CEUS 48 h after irreversible electroporation showed the highest correlation with the pathologic ablation zone size (length: r = 0.909, width: r = 0.942, p < 0.001), whereas ultrasound measurements showed the lowest correlation (length: r = 0.676, width: r = 0.842, p < 0.001).

**Conclusions:**

Compared with conventional ultrasound, CEUS can accurately measure the dimension of the ablation area, especially 48 h after irreversible electroporation.

## Introduction

Percutaneous thermal-based ablation methods, such as radiofrequency ablation and microwave ablation, have been widely used for the minimally invasive treatment of tumors in the liver, kidneys, and other organs. However, these techniques could have limited effect on lesions adjacent to important organs due to the heat sink effect, which weakens the tumoricidal effect.^1^ Irreversible electroporation is an alternative treatment for these lesions.^2,3^

Irreversible electroporation is a novel type of localized tissue ablation that involves the targeted delivery of electrical pulses to create nanoscale pores in cell membranes.^4^ This technique causes irreversible disruption of cell membrane integrity and subsequent cell death at sufficient electrical doses. Previous studies have utilized single-needle devices for irreversible electroporation delivery in porcine livers.^5–7^ Moreover, there are multiple studies describing the characteristics of irreversible electroporation ablation in porcine livers.^8,9^

Contrast-enhanced ultrasound (CEUS) allows for continuous real-time observation of blood flow of the hepatic artery, portal vein, and tissue perfusion.^10^ Preliminary animal and clinical studies have reported the feasibility of using CEUS to assess ablation areas. CEUS can be used to evaluate the size and characteristics of the ablation zone.^11^ However, limited data are available on the CEUS imaging characteristics and dimensions of ablation lesions after irreversible electroporation using bipolar electrodes.

The aim of this study was to evaluate the ultrasound and pathological features of the ablation area in porcine livers, immediately after, and 48 hours post-irreversible electroporation ablation using bipolar electrodes. The study specifically aimed to define the effective treatment boundary and assess changes in lesion size on ultrasound and CEUS.

## Materials and methods

### Animal model

All animal experiments were approved by the Laboratory Animal Ethics Committee of the First Affiliated Hospital, Zhejiang University School of Medicine and conducted in accordance with relevant ethical guidelines for animal research (IACUC-20231023-23). Animal husbandry and all experimental procedures were carried out in accordance with institutional guidelines. Three pigs weighing between 65–70 kg were used in this study. Anesthesia was induced with an intravenous bolus of propofol (2 mg/kg). Analgesia was provided and anesthesia was maintained with a continuous fentanyl infusion (1 mg per 1.5 h). Heart rate and rhythm were continuously monitored, and physiological parameters were monitored throughout the procedure in accordance with standard laboratory animal care guidelines.^12–14^ To minimize involuntary muscle contractions associated with irreversible electroporation, cisatracurium (0.2 mg/kg) was administered intravenously approximately 2 min prior to pulse delivery to achieve neuromuscular blockade.^15–17^ The liver was exposed through bilateral subcostal incisions. The animals received analgesic treatment (4 mg/kg, intramuscularly) daily for 2 d to manage postoperative discomfort following the irreversible electroporation procedure.

### Irreversible electroporation

Irreversible electroporation was conducted using a single bipolar 18-gauge electrode (Curaway, China), including three configurations (length combinations). All experiments used custom-designed coaxial bipolar needle electrodes. These electrodes consisted of a front electrode needle and a rear electrode needle, coaxially arranged and electrically isolated by an insulating element. The electrode rod diameter was 18 gauge (18 G), with a triangular distal tip to facilitate tissue penetration. All electrodes used the same material, outer diameter (18G), and tip geometry; the only differences between configurations were the axial length of the exposed conductive section and the length of the insulating spacer ring.

**FIGURE 1.**
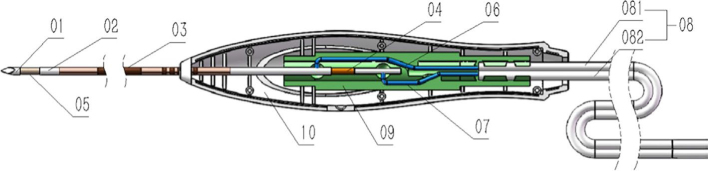
Schematic of the bipolar IRE needle electrode device 01. Front electrode needle; 02. Rear electrode needle tube; 03. Outer insulating tube; 04. Inner insulating tube; 05. Insulating spacer ring; 06. Rear electrode adapter wire; 07. Front electrode adapter wire; 08. Parallel double-strand cable; 09. Needle tube fixing component; 10. Handle housing.

The electrode needle dimensions were 3 mm–10 mm–3 mm, 6 mm–6 mm–6 mm, and 3 mm–8 mm–3 mm. The middle value denotes the length of the insulating spacer ring, whereas the two side values denote the lengths of the exposed electrode segments. Under identical pulse settings and placement procedures, the 3–8–3 mm configuration produced the largest ablation area and was therefore selected for all subsequent in vivo experiments. The remaining configurations (3–10–3 mm and 6–6–6 mm) were used only for the initial geometric exploration. The electrode used in the study consisted of a handle, a needle body, and an insulating spacer ring (Figure 1). The handle was made of acrylonitrile butadiene styrene plastic, the insulating spacer ring was made of polytetrafluoroethylene, and the needle body was made of stainless steel. The working tip of the electrode needle contained an emitter and a return electrode, and only one needle was needed for ablation. Energy deposition ranged from 1500 to 2800 V (pulse length: 100 μs; number of pulses: 40–100).

The needle placement was performed percutaneously under ultrasound guidance (Mindray M9 Ultrasound Machine, China) by an experienced abdominal interventional radiologist with over 20 years of expertise. Eight non-overlapping irreversible electroporation sites were created on each pig liver. To avoid overlapping ablation areas and limit cumulative parenchymal damage, the minimum distance between any two sites was at least 3 cm. The sites were distributed within the liver parenchyma (rather than concentrated in a single region).

### Ultrasound

Ultrasound was utilized during the operation, as well as immediately and 48 h after irreversible electroporation, to assess imaging characteristics and dimensions of the ablation area. The ultrasound machine (Mindray M9, China) equipped with a 7–12 MHz linear probe was used in our study. CEUS was performed immediately and 48 h after irreversible electroporation to evaluate the ablation effect. All CEUS examinations were performed using intravenous Sonazoid (GE Healthcare, Oslo, Norway) as a contrast agent. Following the injection of 1 mL of Sonazoid, 5 mL of 0.9% saline was administered. A dual-B-mode image was acquired immediately after injecting the ultrasound contrast agent, with the timer started simultaneously. Three phases were defined as follows: the arterial phase (15–30 s), the portal venous phase (31–120 s), and the late phase (121 s and later). The ablation zone was continuously observed for 10 min. All examination images were digitally recorded and stored on the hard disk of the US scanner for subsequent analysis.

### Histologic analysis

The pigs were euthanized 48 h after irreversible electroporation according to the approved institutional animal protocol. Following euthanasia, the livers were harvested and sliced into approximately 5-mm-thick sections parallel to the electrode tract. All liver specimens underwent H&E staining, and a subset was additionally stained with terminal deoxynucleotidyl transferase dUTP nick end labelling (TUNEL) for apoptosis analysis. The histopathological specimens were evaluated by an attending pathologist who delineated the boundaries of each inner zone.

### Statistical analysis

The correlation between the ablation area size, as measured in the ultrasound and the ablation zone size determined pathologically, was assessed using the correlation coefficient. Interobserver variance between one researcher and other physicians, as well as intraobserver variance in measuring the irreversible electroporation ablation zones, on both ultrasound images and histological slides were evaluated using intraclass correlation coefficients. Statistical significance was set at p < 0.05. The calculations were conducted using SPSS Statistics for Windows (Version 23.0).

## Results

**FIGURE 2.**
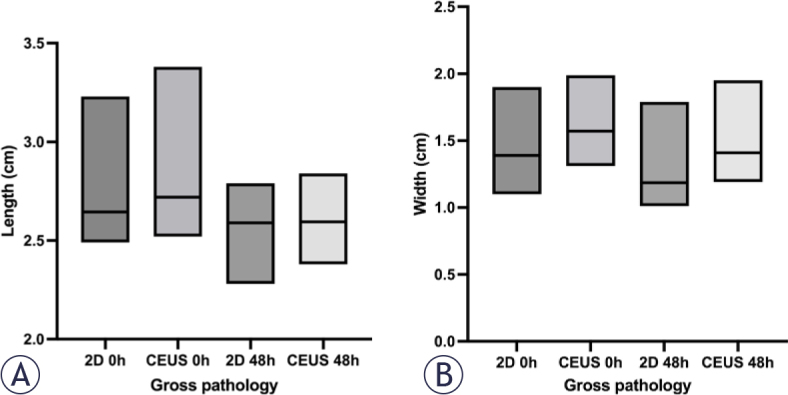
**(A)** Comparison of measurements of the length of the ablation zone (cm) by ultrasound, CEUS, and Gross Pathology. **(B)** Comparison of measurements of the width of ablation by ultrasound, CEUS, and Gross Pathology 2D 0 h: Immediately after irreversible electroporation by two dimensional ultrasound; 2D 48 h: 4 8h after irreversible electroporation by two dimensional ultrasound; CEUS 0h: Immediately after irreversible electroporation by CEUS; CEUS 48 h: 48 h after irreversible electroporation by CEUS.

All irreversible electroporation ablation procedures were successfully accomplished, without immediate complications. A total of twenty-four ablations were created in three swine. The dimensions of the ablation area on ultrasound, CEUS, and histopathology are shown in Figure 2. The dimensions of the ablation area on gross pathologic assessment were as follows: 2.6 ± 0.2 cm in length (range: 2.3–2.9 cm) and 1.4 ± 0.2 cm in width (range: 1.1–1.9 cm).

### Ultrasound findings

**FIGURE 3.**
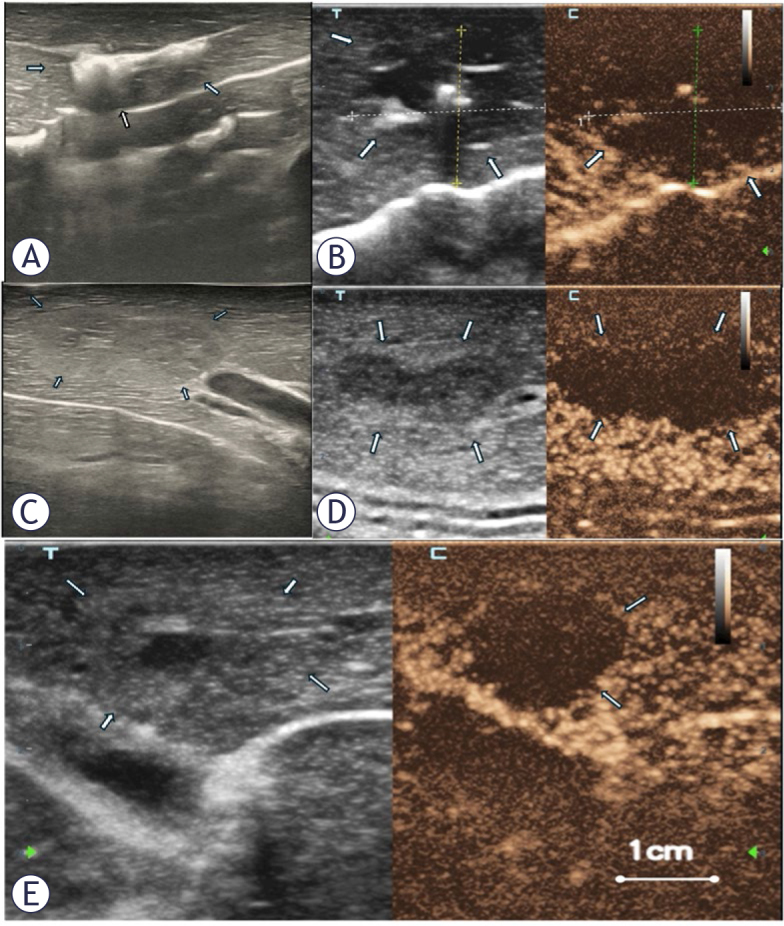
Ultrasound and CEUS findings after irreversible electroporation ablation. A scan obtained during ablation **(A)** confirms that the position of the electrode is appropriate. Scan obtained immediately after irreversible electroporation application **(B)** showing a hypoechoic ablative zone with well-demarcated margins surrounding the electrode. It appears as an avascular area in the arterial phase immediately after ablation. Scan obtained 48 h after irreversible electroporation **(C)** showing that the echogenicity of the central ablation zone increases, whereas the surrounding area is a ring-shaped hyperechoic band. Long axis view **(D)** and short axis view **(E)** showing that the ablation zone appears as a hypo-enhanced area in the arterial phase, and there is internal enhancement within the ablation zone 48 h after irreversible electroporation ablation.

Real-time ultrasound showed that the liver tissue around the electrode needle was uniformly hypoechoic, and the hypoechoic area continued to increase in size over time. CEUS revealed no enhancement in the ablation area, with a clearly outlined boundary immediately after irreversible electroporation. The diameter measured 2.7 ± 0.2 cm in length (range, 2.5–3.2 cm) and 1.5 ± 0.2 cm in width (range, 1.1–1.9 cm) on ultrasound immediately after irreversible electroporation. Conversely, the diameter measured 2.8 ± 0.2 cm in length (range, 2.5–3.4 cm) and 1.6 ± 0.2 cm in width (range, 1.3–2.0 cm) on CEUS. The echogenicity of the central zone exhibited heterogeneity, while the border of the hyperechoic band appeared blurred 48 h after irreversible electroporation on ultrasound. The treatment zones had a mean value of 2.6 ± 0.1 cm in length (range, 2.3–2.8 cm) and 1.3 ± 0.2 cm in width (range, 1.0–1.8 cm), including the rim. Colour Doppler Flow Imaging detected blood flow signals within the ablation lesion. The ablation zone displayed centripetal internal enhancement, particularly evident in the arterial phase on CEUS 48 h after irreversible electroporation. The mean diameter of the ablative zone was 2.6 ± 0.1 cm in length (range, 2.4–2.8 cm) and 1.5 ± 0.2 cm in width (range, 1.2–1.9 cm) at this time. The temporal evolution of the irreversible electroporation ablation area on ultrasound and CEUS is shown in Figure 3.

### Pathologic findings

#### Gross pathologic examination

The ablative areas of the specimens harvested 48 h after irreversible electroporation appeared as two main zones: an inner dark red zone (areas surrounding the electrode) and an outer light red zone. The traversing vessels and bile duct walls within the ablation zone appeared intact macroscopically.

#### Histologic examination

The pathological results revealed that the liver tissue was clearly divided into different zones. Haemorrhage and necrosis of liver cells were observed in the central liver lobules. Hepatocytes in the transition zone exhibited significant degeneration, accompanied by the proliferation of fibrous tissue and infiltration of inflammatory cells. Although the structures of the interlobular bile ducts, interlobular veins, and interlobular arteries in the portal area persisted, they were accompanied by cell degeneration. Mild cholangiocyte degeneration was observed outside the transitional zone. The central area of the ablative zone contained a higher concentration of blood cells than the peripheral area.

**FIGURE 4.**
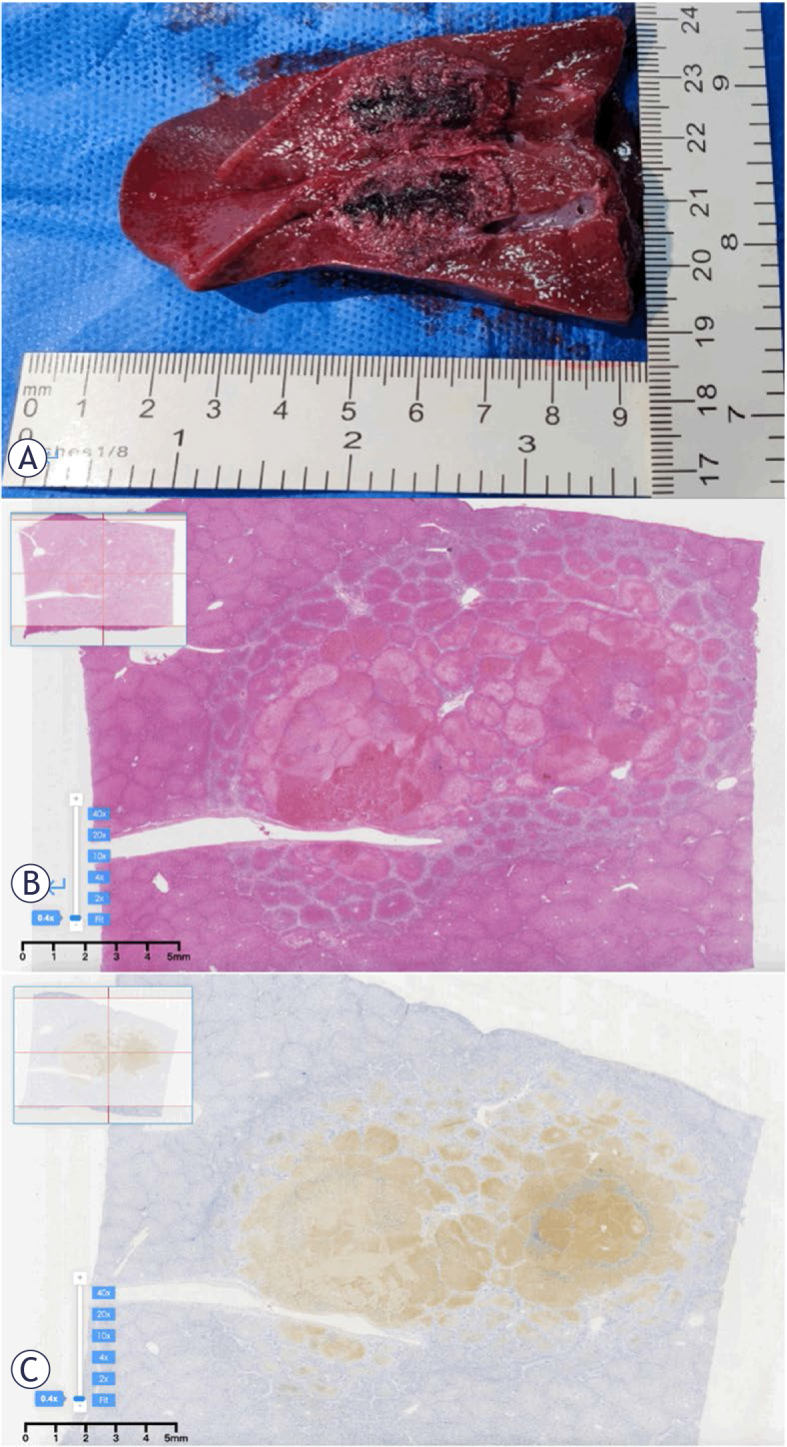
Gross specimen and histopathology of tissue zones of irreversible electroporation-ablated lesion. **(A)** Cut gross specimen. **(B)** Photomicrograph of a specimen from specimens obtained 48 h after irreversible electroporation. **(C)** Representative TUNEL staining of the irreversible electroporation ablation zone showing extensive TUNEL-positive cells.

The histopathological evaluation identified two distinct tissue sublayers: the inner and transitional zones. The inner and transition zones showed positive TUNEL staining, indicating apoptotic cell death and the absence of microscopically viable hepatocytes (Figure 4).

### Correlation between US and pathologic findings

The ablation lesion size measured on CEUS 48 h after irreversible electroporation showed the highest correlation with the pathologic ablation zone size (length: r = 0.909, width: r = 0.942, p < 0.001). Ultrasound and CEUS performed immediately after irreversible electroporation exhibited similar correlations with the size of the pathological ablation lesions. The correlation between ultrasound and the size of pathological ablation lesions was the lowest 48 h after irreversible electroporation (length: r = 0.676, width: r = 0.842, p < 0.001). The scatter plots showed the ablation zone size on histopathology compared with that of ablation zone on ultrasound and CEUS (Figure 5). Intraclass correlation coefficients ranged from 0.947 to 0.991, indicating good inter- and intra-observer reproducibility between ultrasound imaging and histological ablation area measurements.

**FIGURE 5.**
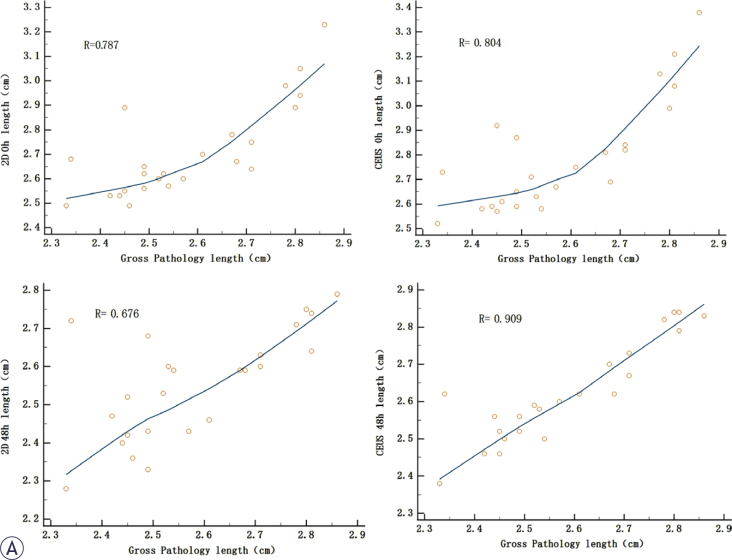
Dimension of the ablation zone on Gross Pathology versus the ablation area measurement on ultrasound and CEUS. **(A)** Correlation between the length of the ablation area with gross pathology.

**FIGURE 5.**
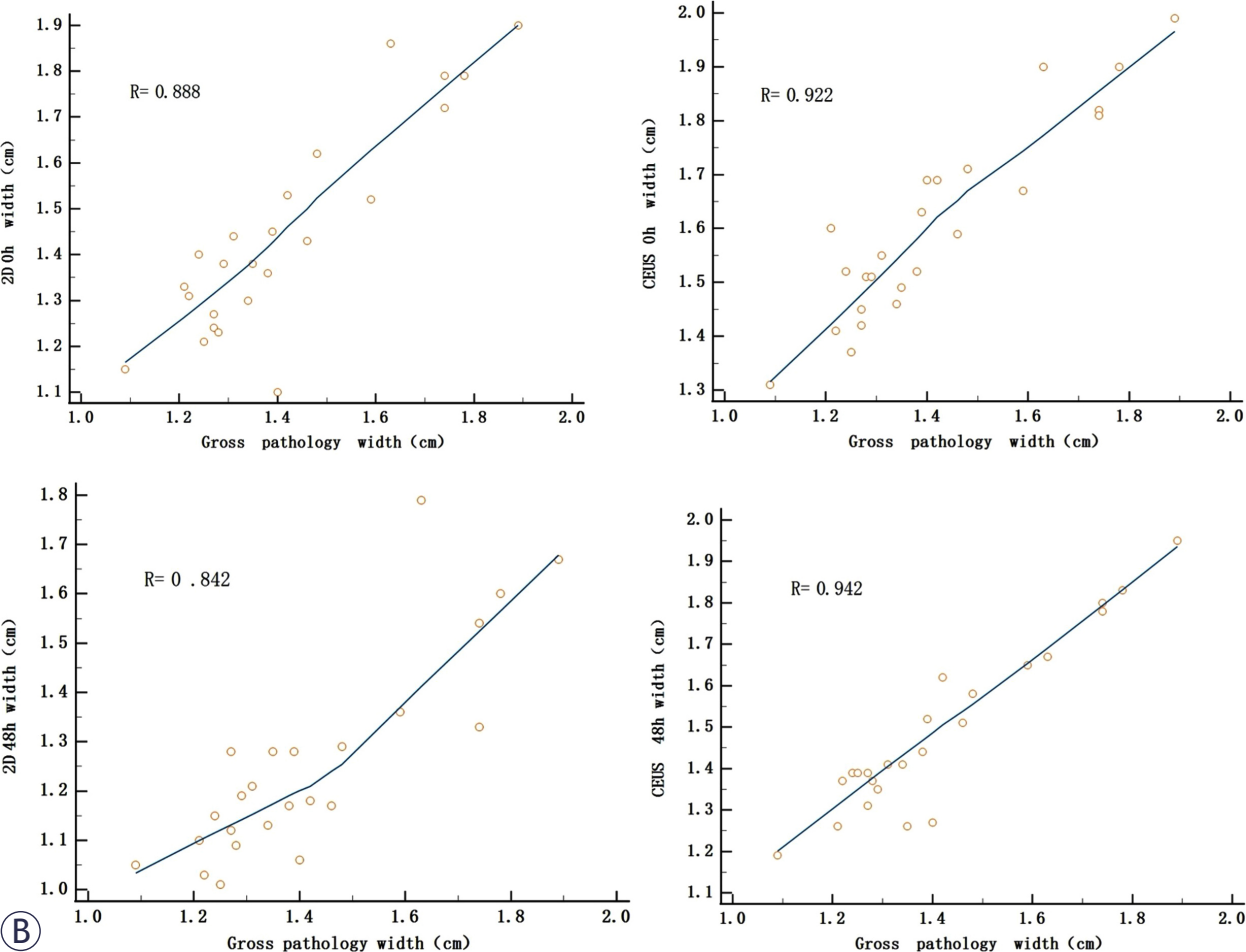
Dimension of the ablation zone on Gross Pathology versus the ablation area measurement on ultrasound and CEUS. **(B)** Correlation between the width of the ablation area with gross pathology.

## Discussion

Irreversible electroporation has garnered increasing interest due to its non-thermal properties, which allow for the ablation of tumors around vital structures. Numerous studies exploring the correlation between radiographic findings and histopathology have enriched our comprehension of the mechanisms of irreversible electroporation ablation.^18–20^ Prior investigations have demonstrated that ultrasound findings following irreversible electroporation application to liver tissue evolve from within seconds to several hours.^8,21,22^ Notably, preclinical animal studies indicate that ultrasound (especially CEUS) can serve as a time-dependent surrogate of electroporation-induced tissue injury by capturing evolving perfusion deficits that ultimately align with gross and histopathologic findings.^23,24^ However, only a few studies have explored the ultrasonographic characteristics of the entire ablation area, encompassing the delineation of effective treatment boundaries and changes in lesion size both on ultrasound and CEUS. Unlike prior porcine studies that focused primarily on the very early (< 2 h) post-IRE window, our inclusion of a 48-h follow-up with direct CEUS–histopathology size comparison clarifies when imaging-based lesion measurements stabilize and highlights CEUS as the modality most consistent with final tissue endpoints.

In animal models, the relationship between ultrasound/CEUS and histopathology after electroporation-based ablation is considered a timevarying perfusion-morphology continuum, rather than a static correlation. A proof-of-concept study of irreversible electroporation (IRE) in porcine liver highlighted that grayscale/contrast-enhanced ultrasound images change rapidly in the early post-ablation period (within hours), and this dynamic change may lead to an overestimation or underestimation of the effective ablation extent (if assessed only at a single time point). These findings underscore the importance of choosing appropriate imaging timing and the value of contrast-enhanced ultrasound in early efficacy assessment.^23^ Our findings are consistent with this concept. Furthermore, contrast-enhanced ultrasound studies in canine spontaneous tumor and mouse tumor models have shown that timedependent reductions in perfusion after treatment are associated with treatment response and tumor growth behaviour, consistent with vascular effects as a surrogate imaging endpoint, and providing a mechanistic explanation for the potential improvement in CEUS and histological consistency at later time points, such as 48 hours.^25,26^

Our study demonstrated sequential changes in ultrasound and CEUS findings over time, ranging from immediately to 48 h after irreversible electroporation. Immediately after irreversible electroporation, the ablation area appeared hypoechoic and well-delineated on ultrasound, with no enhancement observed on the CEUS images. Lee *et al*. reported ultrasonographic findings similar to those in our study in 55 ablation areas, showing that the immediate hypoechoic appearance of the treated area transformed into hyperechoicity at 24 h.^27^ The hypoechoic observation immediately after ablation was attributed to congestion and oedema, while the subsequent hyperechoicity was associated with an inflammatory response and increased immune cell infiltration in the ablated area. Radiologic-pathological correlation studies indicated red blood cell infiltration at the peripheral edges of the ablation zone compared to the central area 116 min after ablation. Peripheral hyperechoicity was attributed to a higher number of red blood cells around the lesion than at the center.

CEUS can better visualize the boundaries of the ablation zone than ultrasound, facilitating measurement 48 h after irreversible electroporation. Consistent with previous studies, the ablation zone measured at 48 h was smaller than the size measured immediately after ablation. The shrinkage of ablation lesions can be attributed to many causes. Irreversible electroporation disrupts microcirculatory perfusion within tissues, and the extent of the microcirculatory defect may represent the size of the ablation zone.^28^ The oedema around the ablation area gradually subsides with time and the lesion volume decreases.^27^ IRE-induced cell death mainly involves apoptosis, not direct death. Apoptosis requires a certain amount of time to complete programmed death and be cleared by macrophages.^29^ Additionally, the electric field edge effect of IRE may cause reversible electroporation of surrounding cells, which will recover or enter apoptosis after 48 h, thereby shrinking the ablation focus boundary.^4^ Previous follow-up studies using MRI and CT have reached similar conclusions.30,31 This phenomenon suggests that the immediate postoperative imaging evaluation may overestimate the actual ablation range, and the final therapeutic effect requires confirmation by combining the follow-up imaging after 48 h.

Although irreversible electroporation is a nonthermal ablation technique, it may still cause thermal coagulation damage due to the Joule heat effect in clinical applications, affecting its advantages of non-thermal properties. Based on previous research, thermal damage caused by irreversible electroporation can be minimized through parameter optimization, electrode needle design optimization, and real-time monitoring. Short and high-frequency pulses can reduce the risk of thermal damage.^4^ Concurrently, the electric field strength needs to be controlled. Electric field strengths that are too high (> 3000 V/cm) may aggravate Joule heating. The field strength distribution can be optimized through data simulation.^32^ Highly conductive materials can reduce resistive heating.^33^ Nanostructured coatings can enhance charge transfer efficiency, thereby reducing the required voltage.^34^ Internally cooled electrodes can reduce interfacial temperatures by ~36%.^35^

This study has several limitations. First, only normal swine liver was studied, and the results may differ in cases of hepatocellular carcinoma or human liver metastases. Moreover, significant differences between normal and cirrhotic parenchyma may exist regardless of the presence of a tumor. Further studies are needed to examine US imaging in liver tumor models, in which changes in blood vessels and extracellular spaces may differ from those in normal tissues. Second, our study assessed imaging-pathology concordance only up to 48 h after IRE, without long-term follow-up. In the liver, electroporation-based therapy has been reported to show acute injury around electrode tracts at 2 days that may remodel into a smaller, predominantly fibrotic scar by 7 days, with relative preservation of major vascular/biliary structures.^36^ In the pancreas, ECT models describe no CT evidence of pancreatitis at early and 7-day assessments, whereas histology at 1 week shows organized fibrosis formation, with occasional transient enzyme elevations likely related to mechanical duct irritation.^37^ Therefore, future longitudinal studies incorporating later imaging and histopathology time points are needed to characterize tissue remodelling beyond 48 h. This may be an important limitation for clinical applications, as most patients do not undergo scans until at least 1 month after irreversible electroporation. Another limitation is the small number of animals and ablation lesions included.

In summary, CEUS performed at 48 h after irreversible electroporation showed the closest agreement with histopathology for estimating the true size of the ablation zone, particularly in the presence of residual lesions after ablation.
